# Measuring Micro-Friction Torque in MEMS Gas Bearings

**DOI:** 10.3390/s16050726

**Published:** 2016-05-18

**Authors:** Xudong Fang, Huan Liu

**Affiliations:** 1 School of Materials Science & Engineering, Georgia Institute of Technology, Atlanta, GA 30332, USA; 2 School of Optoelectronic Engineering, Xi’an Technological Univeristy, Xi’an 710032, China; liuhuan360@163.com

**Keywords:** micro-friction, force sensor, torque, gas bearing

## Abstract

An *in situ* measurement of micro-friction torque in MEMS gas bearings, which has been a challenging research topic for years, is realized by a system designed in this paper. In the system, a high accuracy micro-force sensor and an electronically-driven table are designed, fabricated and utilized. With appropriate installation of the sensor and bearings on the table, the engine rotor can be driven to rotate with the sensor using a silicon lever beam. One end of the beam is fixed to the shaft of the gas bearing, while the other end is free and in contact with the sensor probe tip. When the sensor begins to rotate with the table, the beam is pushed by the sensor probe to rotate in the same direction. For the beam, the friction torque from the gas bearing is balanced by the torque induced by pushing force from the sensor probe. Thus, the friction torque can be calculated as a product of the pushing force measured by the sensor and the lever arm, which is defined as the distance from the sensor probe tip to the centerline of the bearing. Experimental results demonstrate the feasibility of this system, with a sensitivity of 1.285 mV/μN·m in a range of 0 to 11.76 μN·m when the lever arm is 20 mm long. The measuring range can be modified by varying the length of the lever arm. Thus, this system has wide potential applications in measuring the micro-friction torque of gas bearings in rotating MEMS machines.

## 1. Introduction

Micro-friction in gas bearings plays an important role in MEMS rotating machines, which cannot be ignored. For MEMS machines, improvement of energy density has been a priority for years. To achieve this goal, extensive research has been focused on the fabrication processes, combustion stability, heat loss and friction in rotation bearings [1,2,3,4,5,6,7]. Particularly, high speed is required for high power density rotors, which exacerbates the problem of lubrication. Micro-gas bearings distinguish themselves with low friction, low wear, little contamination and weak adhesion as well as adaptability under various work conditions, when compared to dry friction bearings and electromagnetic bearings. The research relating to micro-gas bearings mainly concentrates on design and fabrication [8,9,10,11], dynamic characterization [12], and slip effects [13,14]. Little is spent on the measurement of friction torque, which is an important parameter. As the friction in gas bearings plays an important role in MEMS rotating machines whose load capacity is very small, it is necessary to measure friction *in situ* to monitor bearing flotation and rotating.

Measurement of micro-friction in rotating MEMS machines is necessary, however, it is still a challenging topic [15,16,17]. MEMS friction and wear have been emphasized because surface forces outweigh the effects of inertia due to the large ratio of surface area to volume. Ku and Guo [18,19] developed a tribometer to measure friction in high sliding MEMS devices lubricated with liquids. Surface-micromachined nanotractor devices were also used to investigate the tribological behavior of linear sliding MEMS devices [20,21]. Compared to the amounts of work on sliding MEMS, the measurement of friction in rotating MEMS is less reported. Several studies can be found in the literature, but most of them are approximations or just numerical models. For example, Zhang *et al.* [22] investigated the performance and stability of gas-lubricated journal micro-bearings in MEMS with a mathematical model and a computational methodology. McCarthy *et al.* [23] indirectly studied dynamic friction of a planar-contact encapsulated micro-ball bearing by monitoring the rotating speed with an optical displacement sensor. Chan *et al.* [24] measured the friction torque of rotating micro-devices incorporating a liquid bearing by assembling permanent magnets onto the silicon rotor and external coils. The friction torque was calculated as balanced with the magnetic moment. One potential issue with this testing system is the magnetic field may be interfered by surroundings. By analysis of the aforementioned literature, there are still obstacles in measuring friction torque in rotating MEMS machines.

One obstacle for applying available sensors to measure friction torque is the work range. The existing devices used to measure friction torque mainly include torque sensors and resistance strain gauges. Generally, the torque sensors can only work precisely when the range is larger than μN·m. For example, MLY Series miniature torque sensors (Magnova, Inc., Pittsfield, MA, USA) and ATF315 low range dis type static torque sensor (Althen Sensors, Kelkheim, Hesse, Germany) have a working range of several mN·m, which cannot be used to measure friction torque in MEMS machines. Work range is not an issue for resistance strain gauges. However, their applications are limited by other factors.

Another issue limiting the application of sensors in measuring friction torque is sensitivity and applied situations. For example, resistance strain gauges, when associated with coupling and supporting bearings, cannot guarantee measurement accuracy if a shaft joint with diameter of 1–2 mm is used to connect the shafts. At the other fixture end of the shaft, new friction induced by the bearing tends to cover the to-be-measured torque. Several systems have been successfully developed for measuring friction torque in bearings. Nevertheless, they are not suitable for the rotating MEMS machines. Bouyer [25] presented an experimental investigation on plain journal bearings during start-up using a U-shape rigid connection to bridge the torque-meter and bushing of the test bearing. It eliminates another fixture bearing, but is not suitable for rotating MEMS machines because there is no bushing in MEMS gas bearings. Kim [15] designed a micro tribo-tester to directly examine the sidewall of the micro-bearings using a micro-turbine, three supporting bearings and a strain gauge. Accuracy of the testing bearing may be affected by new friction torques possibly introduced by the additional supporting bearings. Beerschwinger [26] investigated the dry sliding and rolling friction of microspheres on aluminum, diamond-like carbon and single-crystal silicon surface. The friction study of microspheres on various surfaces was suitable for design of micro-bearing used in micro-motors. McCarthy [23] established an empirical power-law model of dynamic friction in micro-ball bearings over operation conditions. Few of the above cases can be used for *in situ* measurement of friction torque in micro gas bearings due to limit of application situations. Particularly, for micro-engine system with gas-bearings, it may encounter jammed bearings resulting from imbalanced gas paths. In such a situation, the characteristic of high overload is indispensable for the sensor to be adopted. One representative example of the ineffectiveness of high-accuracy force sensor is the Femto-tool sensor FT-S1000 [27]. Its force range of ±1000 μN, gain of 500 μN/V and output signal of 0–5 V meet the measurement requirements and are in the high-sensitivity range. The only limitation is the 300% full-scale overload protection, which is not sufficient for possibly jammed situations. Therefore, design and fabrication of measurement devices for micro-friction torque in MEMS gas bearings is still in urgent need.

Through analysis of the literature, it is reasonable to use a force sensor to detect the friction torque. However, for measuring the friction torque in rotating MEMS gas bearings, the sensor adopted must have good sensitivity, as the friction torque is usually very small. Song *et al.* [28] designed a self-decoupled four-degree-of-freedom force sensor with cross-beam structure, which was easy to fabricate and had good sensitivity. Ma and Qin [29,30] performed finite-element analysis of such cross-beam structures to explain the mechanism of decoupling in detail. Some more tri-axial force sensors using cross-beam structure were designed and fabricated using the piezoresistive principle by doping silicon strain gauges [31,32]. This method is convenient and also useful for miniaturizing sensors that can be applied in more applications, such as biomechanical measurements. The decoupled forces were detected by combing responses from piezoresistors obtained by ion implantation in the high aspect-ratio cross-beam structure [33]. Thus, the cross-beam structure and excellent self-decoupling property can be referred in designing a force sensor for measuring friction torque in MEMS machines with gas bearings.

In this paper, a new system was developed for *in situ* measurement of micro-friction torque produced in the start-up and ready stages of gas bearings in micro-engine systems. With utilization of sensitive and accurate MEMS micro-force sensor, the friction torque can be quantitatively characterized. The friction was converted as a force by a silicon lever arm, which can be detected with the sensor. The merit of this system was the leverage effect. With installation of the sensor on a rotary table to ensure the sensor probe perpendicular to the lever arm, the micro-friction torque can be transmitted out and precisely measured by the force sensor.

## 2. System Design

**Figure 1:**
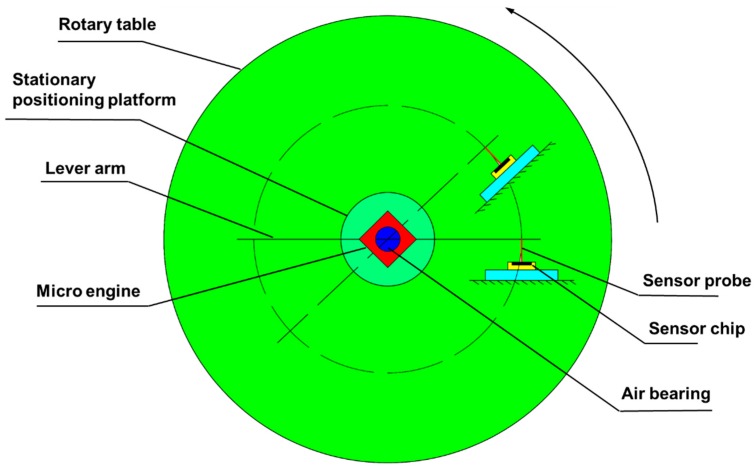
Schematic of the proposed measurement system.

The schematic of the proposed measurement system is shown in Figure 1. The electrically-driven ultra-high precision rotary table provides drive force for the whole system. A designed micro-force sensor is fixed on the table with a horizontally aligned probe. A micro-engine is installed in the central hole of the rotary table with a positioning platform using gas passages for the bearings. Rotation is transmitted to the shaft of the gas bearing by the sensor probe and a silicon lever arm. The cuboid-shape lever arm keeps perpendicular contact with the sensor probe. When the sensor starts to rotate with the table, the lever arm is pushed by the sensor probe and consequently rotates with the engine rotor. According to torque equilibrium, for the lever beam, the friction torque generated by the gas bearing should be balanced by the moment of the pushing force from the sensor probe. Hence, the friction torque can be calculated as a product of the pushing force and the length of the lever arm.

**Figure 2:**
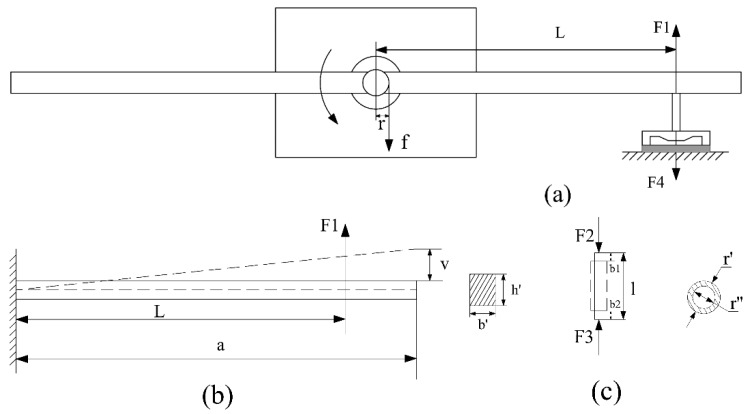
(**a**) Schematic of the measurement mechanism by torque balance; (**b**) Applied force and induced deflections of the lever arm; (**c**) Applied force and induced deflections of the sensor probe.

The fundamental principle applied in the platform design is torque balance. Force transmission in the lever arm system is completed with two beams. One beam is bended and the other one is compressed in the measurement process. Figure 2a describes the force transmission on the lever arm, and that on the sensor probe before the gas bearing starts to rotate. In Figure 2b, the rigid lever beam is indispensable for force transmission. The to-be-measured friction is transmitted through the lever arm, expressed as F1. Simultaneously, the sensor probe receives counterforce of F1, named F2. The quasi-static elastic force F3 is the counterforce of F4, which is applied on the proof mass of the sensor. Torque balance in the system can be expressed with the following equation:

(1) T=f·r=F1·L

In Equation (1), *T* is friction torque generated by the gas bearing, *f* is friction in the gas bearing, and *r* is radius of the rotor. *L* is the distance from the sensor probe tip to the centerline of the bearing. The induced deflections of the lever arm and deformation of the sensor probe can be calculated as follows:

(2) $$v=\frac{2F_{1}L^{2}(3a-L)}{Eb\prime {h\prime }^{3}}$$

(3) $$b=b_{1}+b_{2}=\frac{F_{2}l}{E\pi ({r\prime }^{2}-{r\prime \prime }^{2})}$$

In Equation (2), *a*, *b’* and *h’* are the length, width and thickness of the silicon beam, respectively. *E* is elastic modulus and *v* is deflection of the silicon beam. In Equation (3), *b* is the total deformation of the sensor probe, which is composed of *b1* and *b2*, which are length reduction from the top and bottom, respectively. *l* is the length of the probe. *r’* and *r’’* are the outer diameter and inner diameter of the probe, correspondingly. 

To obtain maximum deflection, F1, F2, F3, F4 are specified as 500 μN. The parameters of the lever arm, namely L, *a*, *b´*, *h´*, are 20, 25, 1 and 0.5 mm, respectively. Calculation shows that the maximum deflection of the lever arm is 1.06 μm that is only 0.424% of the length of the lever arm. The maximum deflection of the probe is 0.12 nm, which guarantees the accuracy of the sensor measurement.

### 2.1. Micro-Force Sensor Design

**Figure 3:**
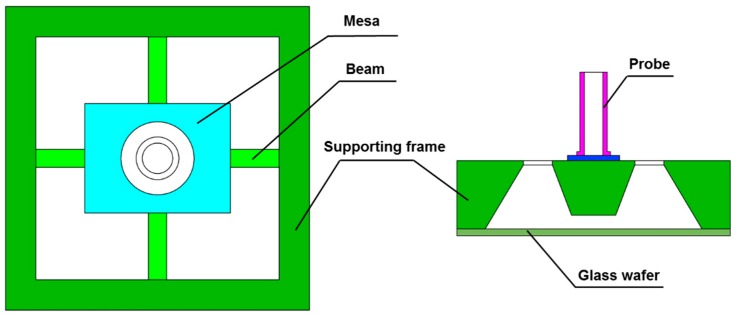
Schematic of the micro-force sensor (not to scale).

The MEMS micro-force sensor is the key component in the measurement system. It works as the intermediate driver of the rotation and the indicator of the friction force, which directly determines the sensitivity and measurement range of the system. High sensitivity and favorable accuracy are necessary for the measurement of μN·m scale friction torque in the gas bearing. A rigid probe and cross beams are utilized as the sensitive element of the force sensor as shown in Figure 3. A stainless steel circular tube coated with a plastic base plate is taken as the probe. Four suspension beams are used to support the central mesa in the flexible sensing structure. To protect the sensor chip under overload situations, a glass wafer is attached onto the bottom of the chip to avoid damage by gas turbulence.

**Figure 4:**
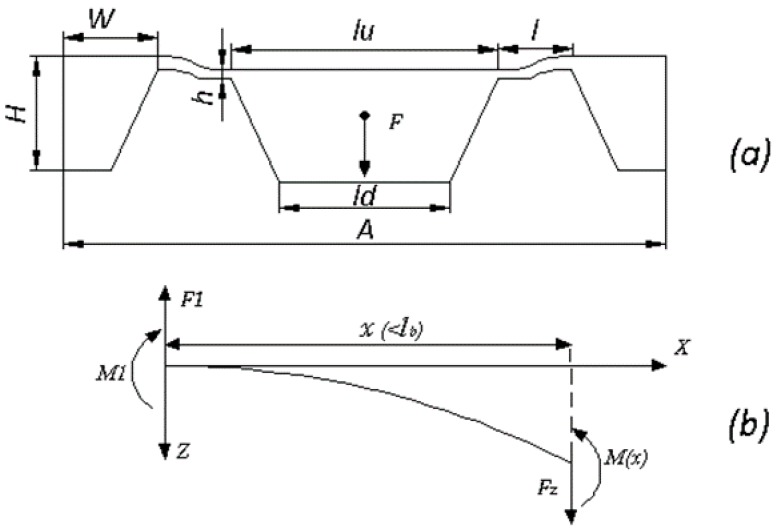
(**a**) Diagrammatic sketch of the chip structure; (**b**) Schematic of applied force and torque balance in the suspending beam.

The chip structure and working mechanism of the suspending beam are shown in Figure 4. According to basic principles of mechanics and small deflections, when an external force *F* is applied, the moment induced at *x* along *X* axis as shown in Figure 4b can be expressed as.

(4) $$M\left(x\right)=F_{Z}x-M_{1}=\frac{F}{4}\left(x-\frac{1}{2}\right)$$

The stress along the beam and the maximum induced stress are deduced as:

(5) $$\sigma =\frac{3lF}{4bh^{2}}\left(\frac{2x}{l}-1\right)$$

(6) $$\sigma_{\max}=\frac{3lF}{4bh^{2}}$$

In Equation (4), *M(x)* is the moment induced at *x*, *F_z_* is the force along *z* direction applied at *x*, and *M*_1_ is the moment at *x* = 0. In Equations (5) and (6), *l*, *b*, and *h* are the length, width and thickness of the suspending beams, respectively. The structural sensitivity, namely the maximum stress under unit force is enlarged if increasing the beam length or diminishing the width. However, determination of the final dimensions is not only based on sensitivity. The limitations from measurement linearity and practical fabrication processes should also be considered. Enough space for the piezoresistors and wires is also required. Taking all these restrictive conditions into account, the parameters are determined as: *l* = 450 μm, *b* = 150 μm, *h* = 35 μm, *lu* = 1920 μm, *ld* = 1400 μm. *lu* and *ld* are the side length of the mesa on the top and bottom surface, respectively. The whole size of the sensor chip is 4 mm × 4 mm × 0.9 mm, and a 4 μm gap between the glass and the bottom of the mesa is formed by ICP etching at the mesa bottom, which serves as the overload protection gap.

Furthermore, a hollow stainless probe is designed. Comparatively light weight and large stiffness make it favorable for uniaxial force transmission. A plastic base plate, coated on the bottom of the stainless probe, is used to paste the probe onto the central mesa. The outer and inner diameter of the probe are 0.45 mm and 0.35 mm, and the length of the probe is specified as 7 mm considering the convenience for fixation in the following tests. Additionally, careful distribution of piezoresistors on the beams is made to eliminate the transverse effect from the mesa and the probe. 

**Figure 5:**
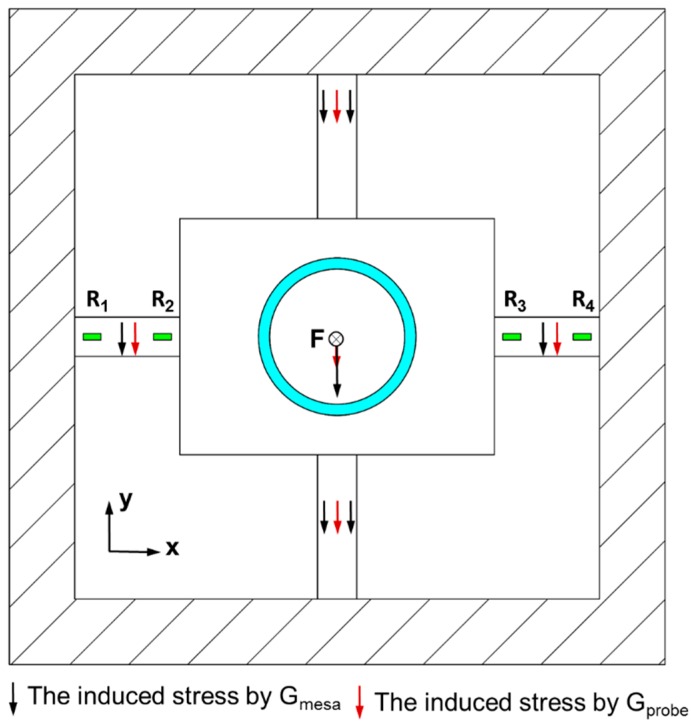
Arrangement of the piezoresistors.

Despite a relatively light probe chosen, there is still unfavorable weight induced by the mesa and the probe if considering the magnitude of the measured force, μN. In the proposed measurement system, the probe is arranged horizontally, and the applied forced *F* is perpendicular to the mesa surface as shown in Figure 5 (pointing into the paper). Since the weight induced stress is concentrated on the beams along Y axis as denoted with arrows in Figure 5, the piezoresistors are positioned on beams along X axis to diminish influence from additional weight.

**Figure 6:**
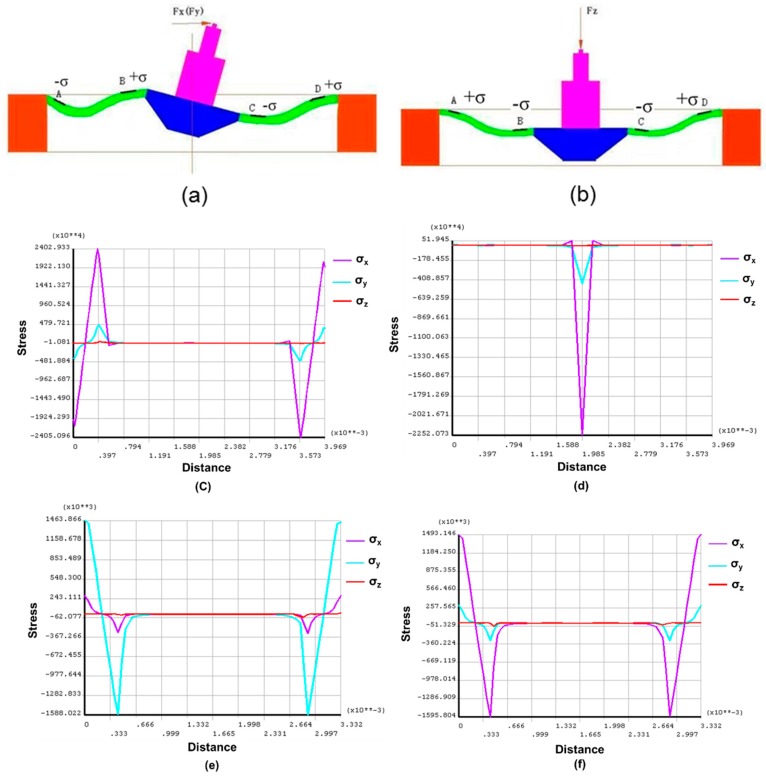
(**a**) Load *F_x_*; (**b**) Load *F_z_*; (**c**) Stress distribution on *x* beams with load of *F_x_*; (**d**) Stress distribution on *y* beams with load of *F_x_*; (**e**) Stress distribution on *y* beams with load of *F_z_*; (**f**) Stress distribution on *x* beams with load of *F_z_*.

In the design of the sensor chip, one key issue to address is decoupling of applied three- dimensional forces. To achieve this goal, in the system design, the silicon beam is required to be perpendicular to the sensor probe to detect only the pushing force. In the sensor design, the decoupling of forces from other directions are also considered. Finite-element analysis was conducted for the sensor chip to effectively utilize the stress distribution in the suspending beams under load. The simulation was also useful to determine connection methods of the piezoresistors to increase sensitivity. With the maximum load 500 μN, Figure 6a shows the stress distribute in two beams (*x*) when a force (*F_x_*) is applied parallel to the beams through the centerline of the probe. x and y directions are identical as that in Figure 5. The stress in the two beams is center-symmetric with identical values but in reverse directions. Similarly, with load *F_z_* applied to the probe, the beams along x and y directions show same deflections as shown in Figure 6b. Positive stress is aligned far from the centerline, while negative stress concentrates near the mesa.

**Figure 7:**
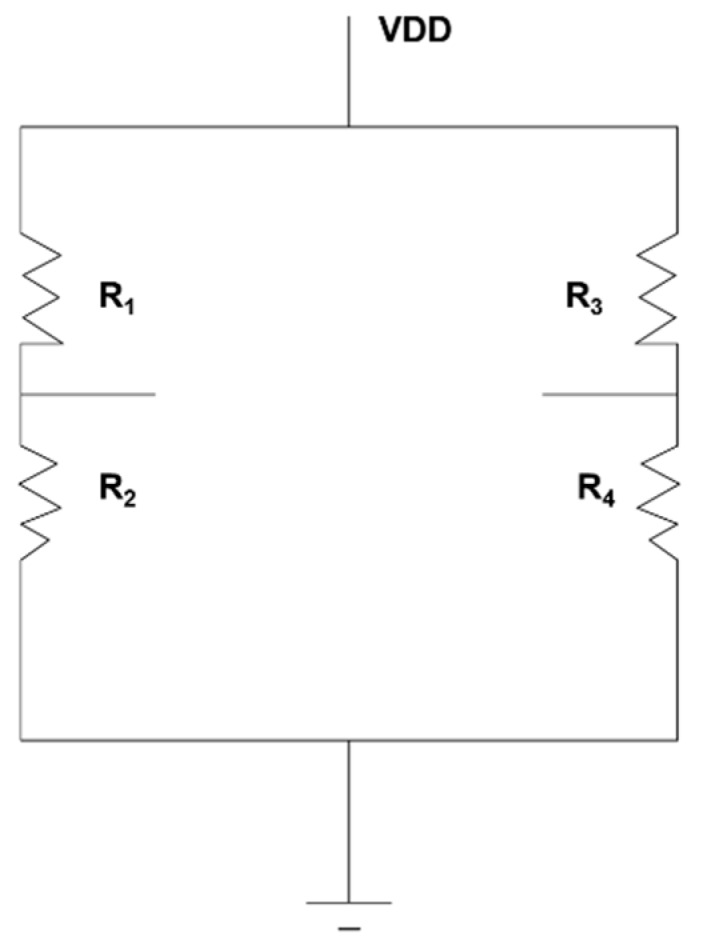
Wheatstone bridge for connecting the piezoresistors.

A quantitative description of stress distribution on the beams are obtained with ANSYS, as shown in Figure 6c–f. With horizontal load *F_x_* applied as in Figure 6a, the stress distribution on the beams (x) is shown in Figure 6c. Comparatively, the stress distribution on the perpendicular beams (y) is described in Figure 6d. It can be obtained that the effect of load *F_x_* on stress distribution of y beams concentrates on the mesa center, where no piezoresistors are aligned. Similarly, the effect of *F_y_* on the stress distribution of x beams is negligible. Thus, the output of *F_x_* and *F_y_* can be decoupled with signals from piezoresistors on their respective beams. Moreover, with perpendicular load *F_z_* applied, the stress distribution on y beams and the stress on x beams are obtained in Figure 6e,f, respectively. It can be observed that the load *F_z_* only significantly affects the stress *σ_x_* on x beams, with negligible effects on *σ_y_* and *σ_z_* on the beams. *σ_x_*, *σ_y_* and *σ_z_* are the stresses along x, y, and z direction, correspondingly. The load *F_z_* has similar effects on stress distribution on y beams due to symmetric structure. Thus, the load *F_z_* can be detected with piezoresistors on either x beams or y beams. While the signals from *F_x_* and *F_y_*, or twisting effects from horizontal forces like that in Figure 6a are ‘noises’ for measurement of friction torque in this work. Thus, the piezoresistors are only distributed on the x beams as shown in Figure 5 and they are connected with a Wheatstone bridge in Figure 7 for decoupling and increasing sensitivity while reducing the manufacturing cost for the chips.

### 2.2. Measurement Platform Design

**Figure 8:**
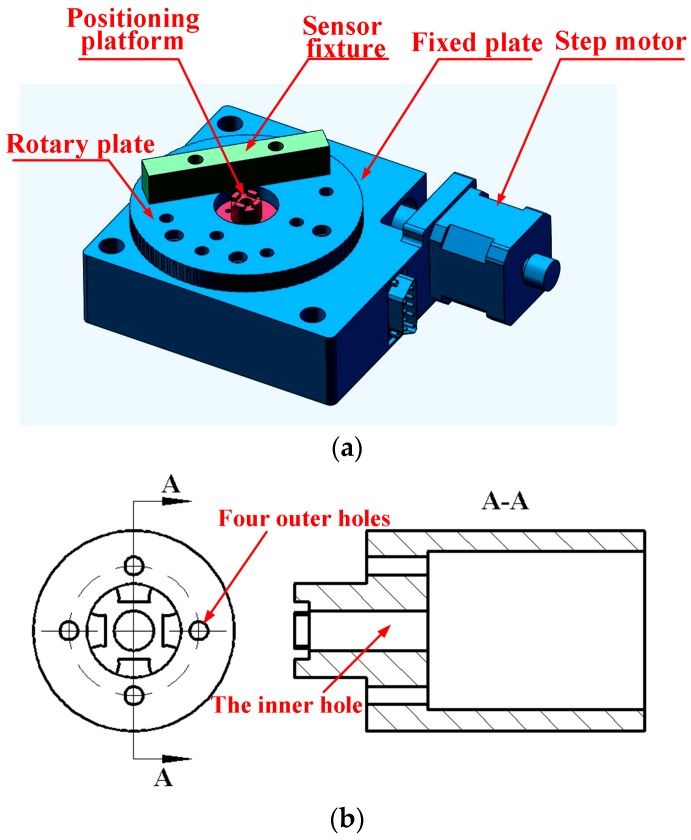
(**a**) Measurement platform; (**b**) Positioning platform.

As shown in Figure 8, the measurement platform for installation of the sensor and bearings is completed based on the ultra-high precision electrically-controlled rotary table. A step motor generates the computer-controlled motion and drives the rotary plate. The whole platform is fixed onto a horizontal stand with the fixed plate. A positioning platform, maintaining the stationary state in the whole measurement, is located in the central hole of the rotary table to fix the tested bearings and provide the gas passage of high purity nitrogen for the gas bearings. The four outer holes are for the pipes leading to an upper journal and thrust bearings. The inner one is for the pipes leading to a journal and thrust bearings at the bottom of the gas bearing. An interference fit is selected for assembly of the positioning platform and the rotary table. The sensor fixture is off-center settled on the rotary plate, and the force sensor is adhered on the fixture with an adjustable distance from the table center.

## 3. System Execution

**Figure 9:**
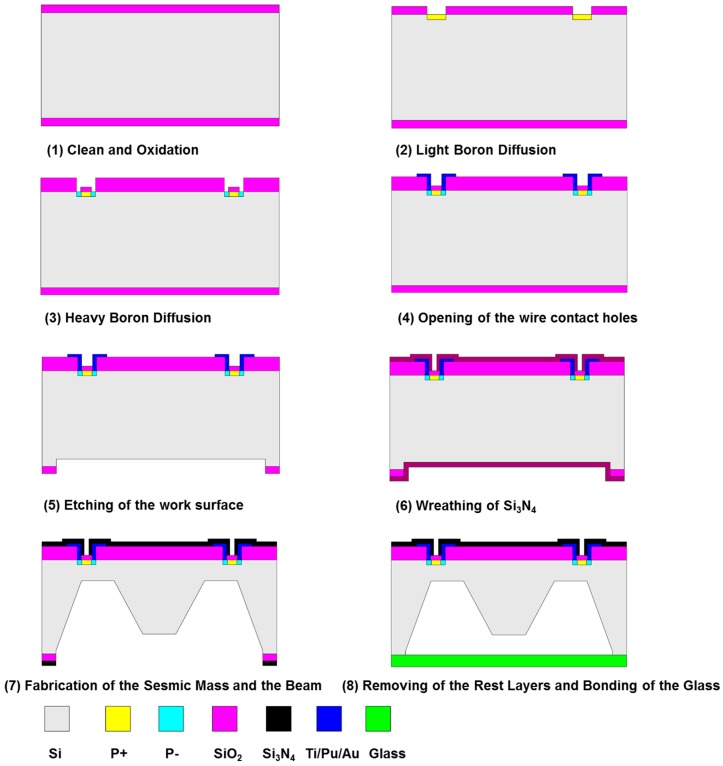
Flow chart of the fabrication process for the sensor chip.

The sensor prototypes were fabricated using KOH etchant and anodic bonding. A double-side polished, n-type, (100) oriented silicon wafer was utilized as the starting material with the fabrication flow sketched in Figure 9. A total of eight masks were necessary, seven for fabrication of the sensing element and one for the metal electrode. The fabrication sequences started with cleaning and oxidizing of the silicon, followed by boron diffusion for resistors, deposition of metal pads and interconnections, etching of the damping gap using ICP technique, preliminary shaping of the proof mass and flexures by KOH etchant, and ended with attachment of Pyrex glass to the silicon substrate by anodic bonding as shown in Figure 9(1–8).

**Figure 10:**
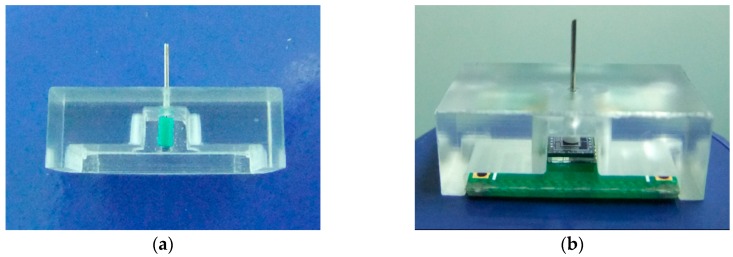
(**a**) Fixture for probe assembly; (**b**) The assembling sensor.

**Figure 11:**
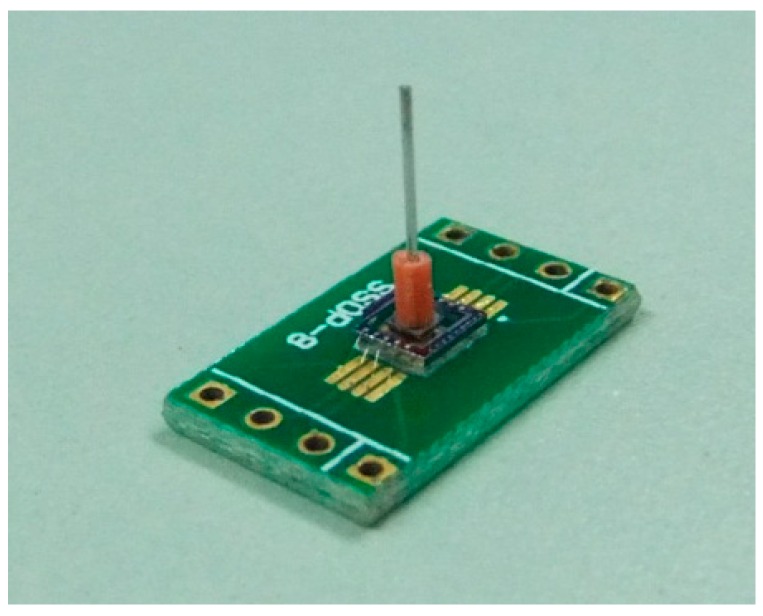
Prototype of the micro-force sensor.

After fabrication of the sensor chip, the probe was attached onto the mesa. A tailor-made fixture, shown in Figure 10, was utilized to guarantee the precision of assembling, keeping the probe vertical to the chip. The fixture had two grooves (one for positioning the PCB and the other for the chip) and a hole (for positioning the probe). The assembling steps were as follows: first, place the sensor chip in the smaller groove; second, dispense the adhesive onto the center of the PCB and place it in the bigger groove to assemble the chip and PCB; and then conduct solidification process at 60 °C for two hours; next adhere the probe onto the mesa by using the glass fixture; finally, bond the micro-sensor chip to PCB with golden leads. The prototype of the sensor was shown in Figure 11.

**Figure 12:**
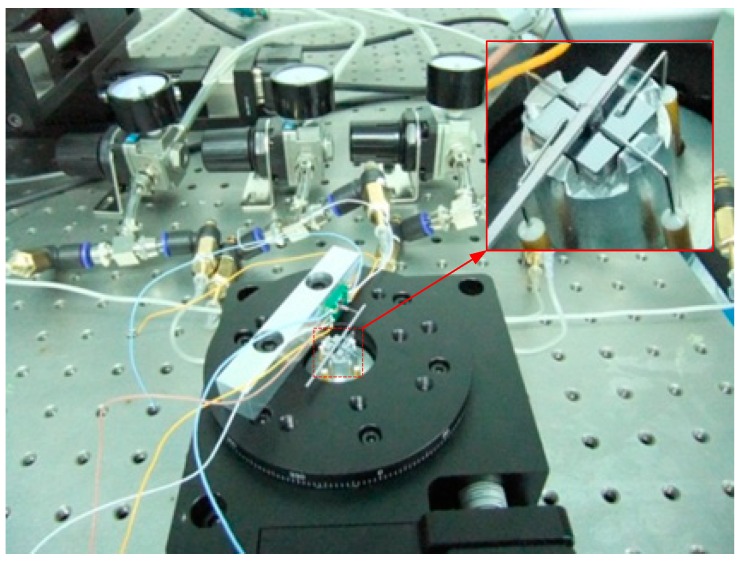
The measurement platform.

The rigid lever arm was fixed to the shaft of the micro-engine along the radial direction and to the probe, transmitting the force from the shaft of the bearing to the senor. Silicon wafer was used to fabricate the lever arm by laser scribing apparatus, and the final size was 50 mm × 1 mm. The sensor fixture was parallel to the lever arm and the sensor probe was perpendicularly in contact with the lever arm. Length of the lever arm can be changed by locating the sensor at different positions and was set as 20 mm in this work. The positioning platform was an aluminum alloy product and fabricated by mechanical processing. The gas pressure for the journal gas bearing was 5 psi. The gas pressure for upper thrust gas bearing was 4.5 psi, while the pressure for the lower gas bearing was set as 4.86 psi to balance the weight of the lever arm, shaft and rotor. The whole finished system is shown in Figure 12.

## 4. Results and Discussions

### 4.1. Calibration of the Force Sensor

**Figure 13:**
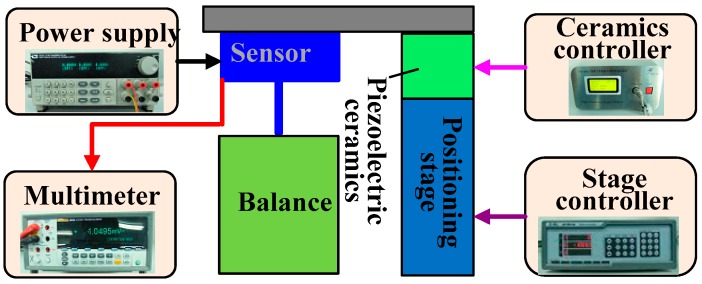
The setup for force sensor calibration.

**Figure 14:**
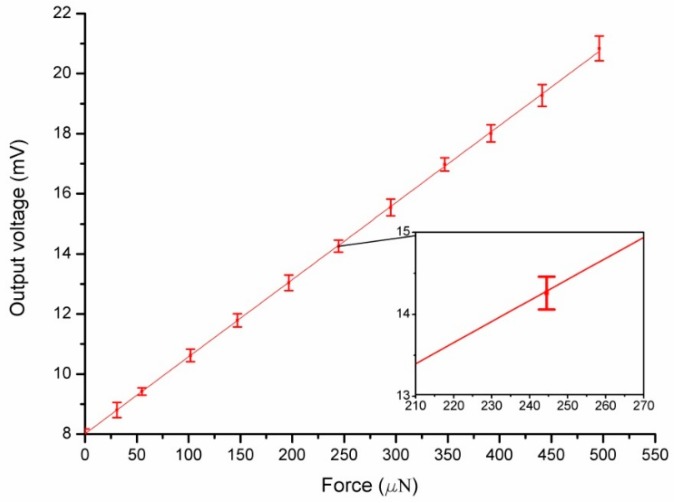
The output voltage of the micro-force sensor *versus* applied force.

Calibration of the designed micro-force sensor was carried out before the system was tested under 5 V DC power supply. The calibration setup (shown in Figure 13) consists of an analytical balance with resolution up to 0.001 mg (about 10 nN), a precise positioning stage and piezoelectric ceramics with resolution up to nanometer scale. When the sensor probe was loaded on the analytical balance, the applied force can be obtained and the corresponding output voltage was measured by a digital multimeter. In this process, a set of forces from 0 to 500 μN were applied to the force sensor. Based on the experimental data, linear fitting was done as shown in Figure 14. The zero drift makes the fitted curve deflected from the origin point because of the weight of the probe and fabrication error of the piezoresistors. More characteristic parameters of the sensor are listed in Table 1. As demonstrated with calibration tests, the cross sensitivity of this sensor is good. The maximum coupled error is 1.24% FS.

### 4.2. Measurement of the Micro-Friction Torque

**Figure 15:**
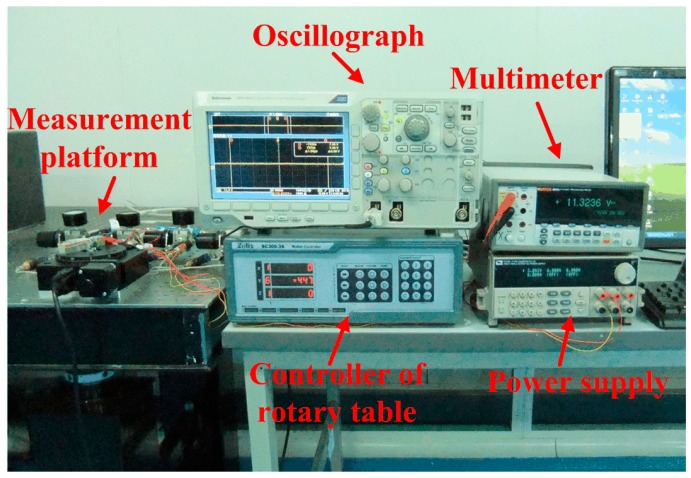
A measurement system for friction torque based on the force sensor.

**Figure 16:**
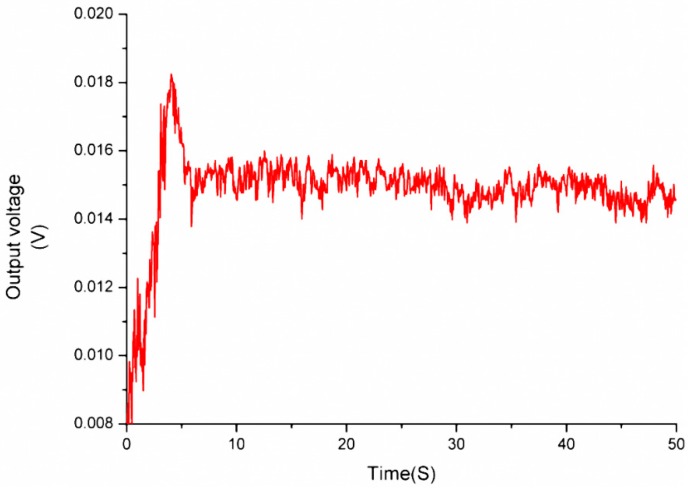
The output voltage of the micro-force sensor under start-up and ready stage.

A measurement system based on the force sensor for measuring friction torque is shown in Figure 15. Running scheme of the rotary table is programmed to rotate about one circle from stillness to uniform rotation of 80/s. The output voltage of the force sensor was recorded by an oscillograph and shown in Figure 16. The biggest starting friction torque occurs when the maximum voltage is output during the starting process. The maximum voltage is 18 mV, and the measured force is 389 μN, which is calculated according to the calibration results in Figure 14. The maximum starting friction torque is 7.78 μN·m, considering the length of the lever arm, 20 mm. The kinetic friction torque is also measured as 5.45 μN·m with the force at the stable stage of 272.5 μN. The large curve fluctuation is possibly caused by unstable gas flow in the gas bearing or gas turbulence in the engine induced by the DRIE-etched rough sidewalls.

## 5. Conclusions

This paper proposed a novel measurement system for micro-friction torque in MEMS bearings and a micro-engine prototype was utilized as the test target. The micro-friction torque was converted into force measured by a micro force sensor when the moment was balanced. The sensor, serving as the key component, was designed, fabricated and characterized. Experimental results demonstrated that the sensor showed good sensitivity and favorable linearity. It has the merits of small work range and high accuracy. With such a sensor, the work range of the designed system is about 0–10 μN·m. The measuring range can be easily modified by varying the length of the lever arm. Therefore, this system has widely potential applications in measuring friction torque of gas bearings in rotating MEMS machines.

## Figures and Tables

**Table 1:** Performance of the micro-force sensor.

Performance Parameters	Value
Sensitivity	0.0257 mV/μN
Nonlinearity	1.28% FS
Zero drift	53.07% FS
Cross-sensitivity with *F_x_*	0.82% FS
Cross-sensitivity with *F_y_*	1.24% FS

## Abbreviations

The following abbreviations are used in this manuscript:

MEMS	MicroElectroMechanical Systems
ICP	Inductively Coupled Plasma
DRIE	Deep Reactive Ion Etching
